# Adipocyte-Mineralocorticoid Receptor Alters Mitochondrial Quality Control Leading to Mitochondrial Dysfunction and Senescence of Visceral Adipose Tissue

**DOI:** 10.3390/ijms22062881

**Published:** 2021-03-12

**Authors:** Clara Lefranc, Malou Friederich-Persson, Fabienne Foufelle, Aurélie Nguyen Dinh Cat, Frédéric Jaisser

**Affiliations:** 1 Centre de Recherche des Cordeliers, Inserm, Sorbonne Université, Université de Paris, Team Metabolic Diseases, Diabetes and Comorbidities, 75006 Paris, France; clara.lefranc@gmail.com (C.L.); fabienne.foufelle@inserm.fr (F.F.); cattuong.ndc@gmail.com (A.N.D.C.); 2 Department of Medical Cell Biology, Uppsala University, 751 23 Uppsala, Sweden; malou.friederich@mcb.uu.se; 3 INSERM Centre d’Investigations Cliniques-Plurithématique 1433, UMR 1116, CHRU de Nancy, French-Clinical Research Infrastructure Network (F-CRIN) INI-CRCT, 54500 Vandœuvre-lès-Nancy, France

**Keywords:** mineralocorticoid receptor, mitochondrial dysfunction, adipose tissue, oxidative stress, senescence, metabolic syndrome

## Abstract

Mineralocorticoid receptor (MR) expression is increased in the adipose tissue (AT) of obese patients and animals. We previously demonstrated that adipocyte-MR overexpression in mice (Adipo-MROE mice) is associated with metabolic alterations. Moreover, we showed that MR regulates mitochondrial dysfunction and cellular senescence in the visceral AT of obese db/db mice. Our hypothesis is that adipocyte-MR overactivation triggers mitochondrial dysfunction and cellular senescence, through increased mitochondrial oxidative stress (OS). Using the Adipo-MROE mice with conditional adipocyte-MR expression, we evaluated the specific effects of adipocyte-MR on global and mitochondrial OS, as well as on OS-induced damage. Mitochondrial function was assessed by high throughput respirometry. Molecular mechanisms were probed in AT focusing on mitochondrial quality control and senescence markers. Adipo-MROE mice exhibited increased mitochondrial OS and altered mitochondrial respiration, associated with reduced biogenesis and increased fission. This was associated with OS-induced DNA-damage and AT premature senescence. In conclusion, targeted adipocyte-MR overexpression leads to an imbalance in mitochondrial dynamics and regeneration, to mitochondrial dysfunction and to ageing in visceral AT. These data bring new insights into the MR-dependent AT dysfunction in obesity.

## 1. Introduction

Obesity and its complications, such as diabetes, dyslipidemia and hypertension, have become a major public health problem worldwide [1]. Obesity is a multifaceted, chronic, low-grade inflammation disease characterised by excess accumulation of dysfunctional adipose tissue (AT). More than just an inert tissue for energy storage, AT is considered as one of the largest endocrine organs in the body and an active tissue in systemic metabolic homeostasis [2]. Indeed, AT can synthesise and release a large number of hormones, cytokines, growth and vasoactive factors, collectively termed adipokines, which influence a variety of physiological and pathophysiological processes. AT dysfunction includes adipocyte hypertrophy and hyperplasia, increased inflammation, impaired extracellular matrix remodelling and fibrosis, together with altered secretion of adipokines and decreased insulin sensitivity [3].

Over the past decade, our group and others have highlighted the important role of aldosterone and its receptor, the mineralocorticoid receptor (MR) in obesity and in metabolic syndrome (MetS) [4,5,6,7], as well as in AT dysfunction [8]. MR expression is increased in the AT of obese patients and animals and targeted overexpression of MR in adipocytes induces a MetS-like phenotype with increased adiposity, dyslipidemia and diabetes [9]. Conversely, inactivation of adipocyte-MR in mice fed a high-fat diet decreases the expression of genes involved in adipogenesis [10]. The ligand activating the MR in adipocytes in vivo is still a matter of debate. Some studies suggest that aldosterone (eventually produced locally) is indeed the natural ligand [11], while others support the idea that glucocorticoids activate adipocyte MR, since the level of the protecting enzyme 11β-HSD2 is so low or absent that glucocorticoid levels are high enough to preclude aldosterone to bind MR [12]. This may be different in disease situations, where expression of either aldosterone synthase or 11β-HSD2 may be induced. In mice with targeted overexpression of MR in adipocytes, we previously reported no difference between aldosterone and corticosterone levels, neither in plasma nor in adipose tissue conditioned medium [13].

Obesity and comorbidities increase levels of reactive oxygen species (ROS) and decrease the antioxidant capacity in AT, leading to oxidative stress (OS) [14,15]. Mitochondria are a major source of ROS in AT and their dysfunction contributes to the pathogenesis of obesity [16]. Our group recently showed the implication of MR activation in AT mitochondrial OS in obese db/db mice [17]. Furthermore, the implication of aldosterone-induced OS and subsequent DNA damage has been established in the liver [18,19].

Mitochondrial integrity is regulated by a number of mechanisms: (i) biogenesis, enabling the synthesis of new molecules of mitochondrial DNA (mtDNA), (ii) dynamics including fusion and fission to shape the mitochondrial network in accordance to the metabolic needs of the cell [20,21] and (iii) mitophagy to eliminate dysfunctional mitochondria. These different processes are designated as mitochondrial quality control (MQC) as they are necessary to maintain a sufficient and efficient pool of mitochondria. This MQC is altered in the AT of obese patients and mice, with decreased biogenesis and fusion and increased mitophagy and fission [22,23]. In HK-2 cells and in the glomeruli of mice and rats, MR-induced OS triggers mitochondrial dysfunction through alteration of mitochondrial respiration, increased fission, and reduced biogenesis [24,25,26]. However, these specific mechanisms in association with MR activation have not been explored in AT yet.

Dysfunctional mitochondria contribute to the ageing process through increased production of ROS. In line with this hypothesis, obese AT displays premature senescence via the activation of the p53-p21 pathway [27,28]. We recently showed that MR overactivation induces mitochondrial dysfunction and AT ageing through activation of the p53-p21 pathway and decreased levels of sirtuins in obese db/db mice [17]. Thus, we seek to better understand the direct implication of adipocyte-MR in these consequences. 

Since (i) adipocyte-MR overexpression induces a MetS-like phenotype; (ii) mitochondrial dysfunction is a main feature of AT dysfunction in obesity; (iii) pharmacological MR antagonism prevents AT mitochondrial dysfunction, OS and senescence in obese db/db mice, we hypothesise that adipocyte-MR activation leads to AT mitochondrial respiratory dysfunction, OS and MQC impairment, as well as premature AT ageing, contributing to obesity-associated metabolic complications. To address this hypothesis, we studied mitochondrial respiration and ROS production, the expression of markers of oxidative damage and of senescence, as well as key pathways of MQC in a mouse model with adipocyte-targeted overexpression of MR. 

## 2. Results

### 2.1. Adipocyte MR Overexpression Induces Adipocyte MR Overactivation

**Figure 1:**
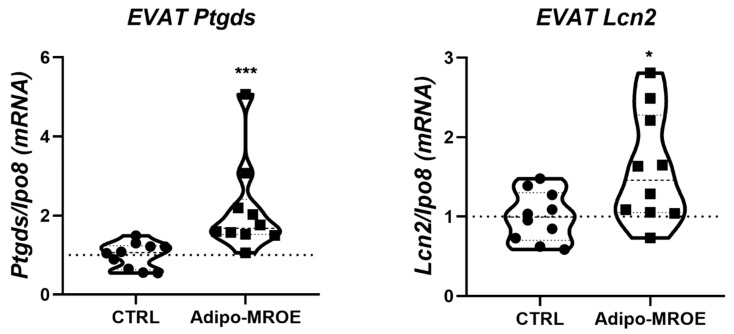
Mineralocorticoid receptor overexpression induces target genes overexpression, showing the existence of mineralocorticoid receptor (MR) overactivation in adipose tissue. *Ipo8*: importin 8; *Ptgds*: Prostaglandin D2 Synthase; *Lcn2*: Lipocalin 2. * *p* < 0.05, *** *p* < 0.001, Adipo-MROE vs. Ctrl. Adipo-MROE: adipocyte-MR overexpression in mice.

We measured the gene expression of both lipocalin 2 (*Lcn2*), an MR target we previously identified in the cardiovascular system [29], and prostaglandin D2 synthase (*Ptgds*), that we identified as a novel MR target specific to the AT in mice and also—importantly—in obese patients [9]. These targets were recently shown to be modulated by MR in the AT of obese patients treated with eplerenone [30]. Gene expression of these two genes was induced in the epididymal visceral AT (EVAT) of Adipo-MROE mice, indicating that MR overexpression in adipocytes is associated with MR overactivation, as shown in Figure 1.

### 2.2. AT MR Activation Induces OS and OS-Induced DNA-Damage

**Figure 2:**
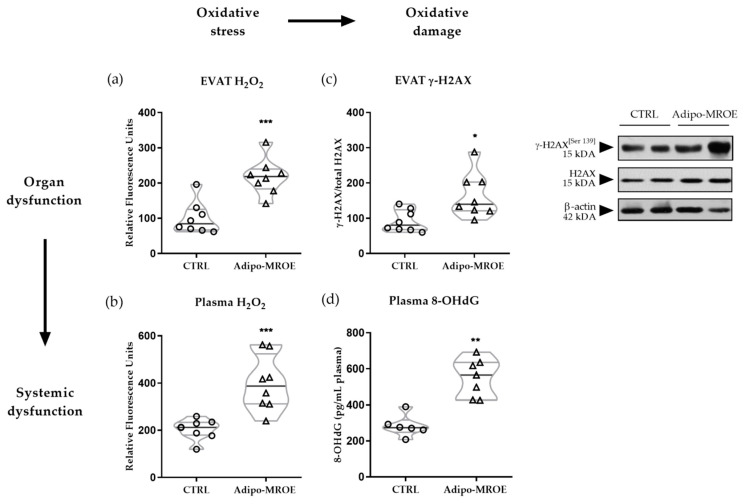
Adipocyte-MR overexpression induces EVAT and plasmatic oxidative stress (OS), with consequent DNA damage in adipocyte-MR overexpressing mice. (**a**,**b**) H_2_O_2_ concentrations, as measured with Amplex Red assay, are increased in EVAT and plasma of Adipo-MROE mice, showing organ and systemic OS. The order of magnitude is the nmol/mg (tissue) or nmol/mL (plasma). (**c**,**d**) EVAT phosphorylation of histone H2A.X—as measured by Western blot—and plasma 8-OHdG levels—as measured by ELISA—are increased in Adipo-MROE mice, showing the existence of oxidative DNA damage. Data are expressed as violin plots with quartiles and individual dots. *n* = 6–8 mice per group; * *p* < 0.05; ** *p* < 0.01; *** *p* < 0.001, Adipo-MROE vs. control. 8-OHdG: 8-Oxo-2’-deoxyguanosine; EVAT: epididymal visceral adipose tissue (AT); MR: mineralocorticoid receptor.

Hydrogen peroxide levels in plasma and EVAT were quantified in the adipocyte-MR overexpressing (Adipo-MROE) mice. Hydrogen peroxide levels were increased in Adipo-MROE mice as compared to controls, indicating increased OS, both in EVAT and in plasma (Figure 2a,b). It is possible to evaluate the consequences of OS on DNA integrity by measuring the levels of histone H2A.X phosphorylation (labelled as γ-H2A.X) in EVAT and of 8-hydroxydeoxyguanosine (8-OHdG) in plasma. Both markers were increased in Adipo-MROE mice, suggesting increased OS-induced DNA damage (Figure 2c,d). These results indicate the implication of adipocyte-MR in local OS and DNA damage, which may contribute to systemic OS.

### 2.3. Adipocyte-MR Activation Disrupts AT Mitochondrial Function

**Figure 3:**
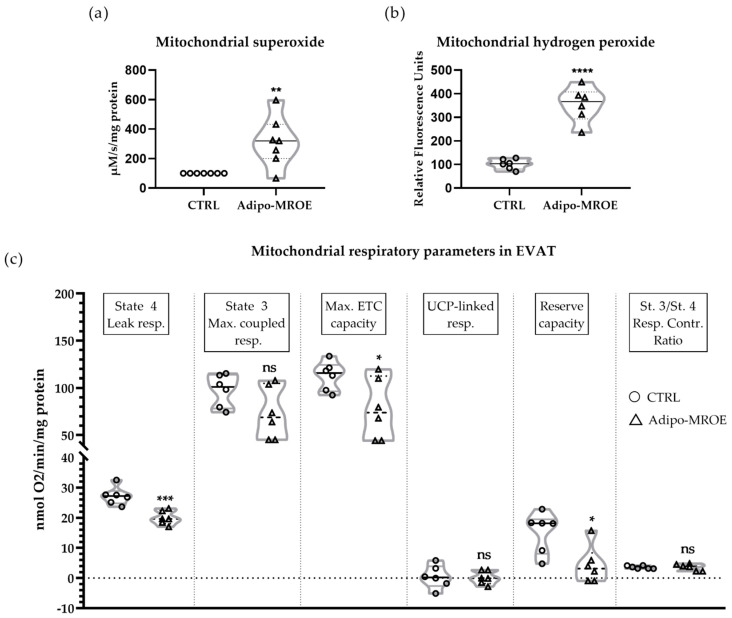
MR-dependent AT OS is linked to mitochondrial OS and altered mitochondrial respiration in the EVAT of adipocyte-MR overexpressing mice. (**a**) EVAT mitochondrial superoxide-as measured by electron paramagnetic resonance (EPR)-and (**b**) the mitochondrial hydrogen peroxide concentration-as measured with Amplex Red assay-are increased in Adipo-MROE mice, showing mitochondrial OS; (**c**) measurement of mitochondrial respiration with the Oroboros O2k-Oxygraph shows decreased state 4 leak respiration and unchanged state 3 maximal coupled respiration in Adipo-MROE mice; leak respiration is not linked to uncoupling proteins (UCP) activation, as shown by the absence of UCP-dependent uncoupling (no effect of the UCP-specific inhibitor GDP) in both groups. Maximal electron transfer chain (ETC) capacity, reflecting ETC function, and reserve capacity-an indicator of energy furniture flexibility-are decreased in Adipo-MROE mice. Data are expressed as violin plots with quartiles and individual dots. *n* = 6 mice per group; * *p* < 0.05; ** *p* < 0.01; *** *p* < 0.001; **** *p* < 0.0001; ns = not significant, Adipo-MROE vs control. Contr.: control; ETC: electron transport chain; EVAT: epididymal visceral adipose tissue; GDP: guanosine diphosphate; MR: mineralocorticoid receptor; OS: oxidative stress; Resp.: respiration; UCP: uncoupling protein.

Mitochondria have been pinpointed in several studies as the origin of MR-dependent OS in the kidney, the heart and AT [17,24,25,26,31,32,33,34]. In our mouse model with AT MR overexpression, both superoxide and hydrogen peroxide production were increased in mitochondria isolated from EVAT (Figure 3a,b). In terms of mitochondrial function, state 4 leak respiration was reduced, while state 3 maximal coupled respiration was not significantly modified (Figure 3c). To explore the origin of reduced leak respiration, we measured the guanosine diphosphate (GDP)-induced oxygen consumption decrease. Indeed, GDP is a specific inhibitor of uncoupling proteins (UCPs), which participate in leak respiration. GDP had no effect in either the control or in the Adipo-MROE mice, ruling out UCP-mediated uncoupling (Figure 3c). Then, we used carbonyl cyanide-4-(trifluoromethoxy)phenylhydrazone (FCCP) to chemically uncouple the activity of the electron transfer chain (ETC) from ATP phosphorylation, in order to measure maximal ETC capacity, which was reduced in Adipo-MROE mice (Figure 3c). The reserve capacity (calculated as the difference between maximal ETC capacity and maximal coupled respiration) was decreased, denoting reduced ability of mitochondria to respond to increased energy requirements (Figure 3c). Finally, we calculated the respiratory control ratio as the ratio between state 3 and state 4 respiration, to estimate the efficiency of coupling and the quality of the mitochondrial preparation, and found no difference between groups (Figure 3c). In summary, MR overexpression in adipocytes is sufficient to induce AT mitochondrial dysfunction, characterised by decreased leak respiration, maximal ETC capacity and reserve capacity, as well as increased mitochondrial OS.

### 2.4. MR-Dependent Mitochondrial Dysfunction Is Due to Impaired MQC

#### 2.4.1. MR Activation Impairs Mitochondrial Biogenesis

**Figure 4:**
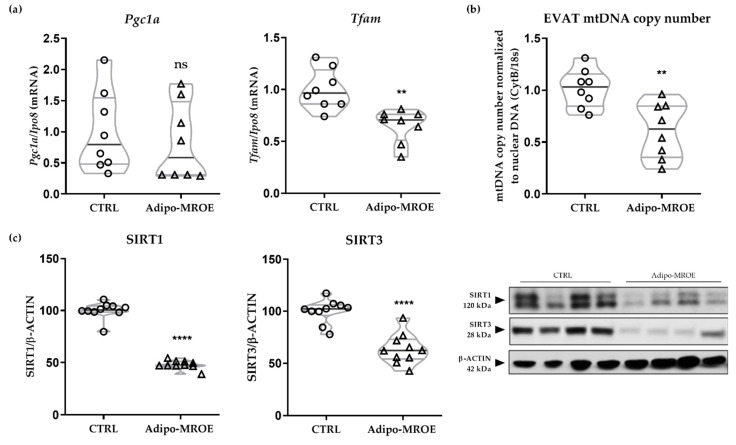
Mitochondrial biogenesis is impaired in the EVAT of adipocyte-MR overexpressing mice. (**a**) mRNA *Pgc1α* levels, as measured by qRT-PCR, are unchanged in the EVAT of Adipo-MROE mice, whereas *Tfam* levels are decreased; (**b**) Consequently, average mtDNA copy number per cell normalised over nuclear DNA copy number is decreased in the EVAT of Adipo-MROE mice. (**c**) Protein levels of SIRT1 and SIRT3 are decreased in the EVAT of Adipo-MROE mice; data are expressed as violin plots with quartiles and individual dots. *n* = 8–10 mice per group; ** *p* < 0.01; **** *p* < 0.0001; ns = not significant, Adipo-MROE vs. control. *CytB*: cytochrome B; EVAT: epididymal visceral adipose tissue; *Ipo8*: importin 8; MR: mineralocorticoid receptor; mtDNA: mitochondrial DNA; *Pgc1a*: Peroxisome proliferator-activated receptor gamma coactivator 1-alpha; SIRT: sirtuin; *Tfam*: mitochondrial transcription factor A.

Mitochondrial biogenesis is regulated by activation of peroxisome proliferator-activated receptor gamma coactivator 1-alpha (PGC1-α), and of its downstream target, the mitochondrial transcription factor TFAM, enabling the synthesis of new molecules of mtDNA [35]. The gene expression of *Tfam*, but not of *Pgc1-α* is decreased in the EVAT of Adipo-MROE mice compared to controls (Figure 4a). Consequently, the number of copies of mtDNA per cell, which is a general marker of mitochondrial fitness, is reduced (Figure 4b). We also analysed the protein expression levels of the sirtuins SIRT1 and SIRT3, regulators of PGC1-α activity [36], which were decreased in Adipo-MROE AT (Figure 4c). We assessed gene expression levels of subunits composing the electron transfer chain (ETC). We observed decreased expression of the NADH:Ubiquinone Oxidoreductase Subunit A9 (*Ndufa9)* and Cytochrome C Oxidase Subunit 5B (*Cox5b*), which are subunits that belong to complexes I and IV (Supplementary Figure S1).

#### 2.4.2. MR Activation Impairs Mitochondrial Dynamics

**Figure 5:**
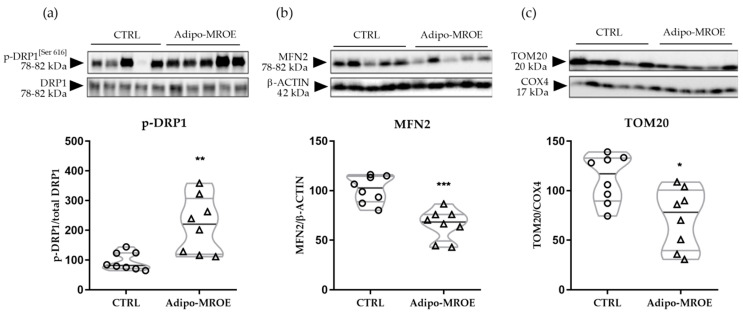
Mitochondrial dynamics are shifted towards fission and mitochondrial degradation, through mitophagy, is impaired in the EVAT of adipocyte-MR overexpressing mice. (**a**) DRP1 phosphorylation and (**b**) MFN2 protein levels, as measured by Western blot, are decreased in the EVAT of Adipo-MROE mice, indicating a shift of dynamics towards fission. (**c**) Protein levels of TOM20 are decreased in the EVAT of Adipo-MROE mice, indicating increased degradation through mitophagy. TOM20 protein levels are normalised by COX4 (mitochondrial protein) to exclude the variation linked to mitochondrial mass. Data are expressed as violin plots with quartiles and individual dots. *n* = 8 mice per group; * *p* < 0.05; ** *p* < 0.01; *** *p* < 0.001, Adipo-MROE vs. control. COX4: cytochrome c oxidase subunit 4; DRP1: dynamin-related protein 1; MFN2: mitofusin-2; MR: mineralocorticoid receptor; TOM20: translocase of outer membrane subunit 20.

Another regulation mechanism of MQC is via fusion and fission [37]. Since sirtuins are known to regulate mitochondrial dynamics [38,39], we studied the expression of key target proteins of this process: dynamin related protein 1 (DRP1) for fission and mitofusine 2 (MFN2) for fusion. Phosphorylation of DRP1 was increased, while expression of MFN2 was decreased in the EVAT of Adipo-MROE mice (Figure 5a,b). This indicates that MR modulates AT mitochondrial dynamics, shifting the balance in favour of fission.

#### 2.4.3. MR Activation Increases Mitophagy

Mitophagy is a mitochondria-targeted autophagy that enables selective degradation of damaged mitochondria. Translocase of outer membrane (TOM) subunit TOM20, an indirect marker of mitophagy, was decreased in Adipo-MROE mice (Figure 5c). This marker is degraded during the mitophagy processes, thus, decreased level suggests an MR-dependent increase in mitophagy in the AT of our model. 

### 2.5. Adipocyte-MR Activation Induces Premature Senescence in AT

**Figure 6:**
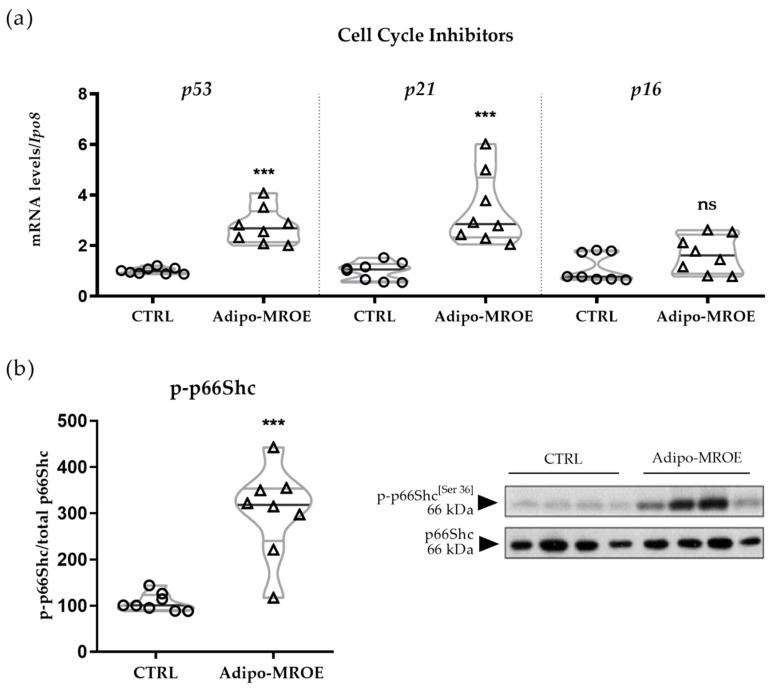
Cellular senescence and OS induced apoptotic pathways are induced in the EVAT of adipocyte-MR overexpressing mice. (**a**) *p53* and *p21* mRNA levels as assessed by qRT-PCR are increased in the EVAT of Adipo-MROE mice, while *p16* mRNA levels are unchanged; (**b**) The phosphorylation of the P21 target p66SHC is increased in the EVAT of Adipo-MROE mice, indicating cellular response to OS. Data are expressed as violin plots with quartiles and individual dots. *n* = 8 mice per group; *** *p* < 0.001; ns = not significant, Adipo-MROE vs. control. *Ipo8*: importin 8.

OS is a major cause of cellular damage that can trigger ageing [40]. To evaluate the consequences of adipocyte-MR overexpression, different markers of cellular senescence, such as the cell cycle inhibitors *p53*, *p21* and *p16*, as well as p66SHC, a downstream target of p53 and p21, were analysed. Expression levels of both *p53* and *p21* mRNA were increased in Adipo-MROE AT as compared to control AT, while *p16* expression was unchanged (Figure 6a). p66SHC phosphorylation was also increased in AT from Adipo-MROE mice as compared to control mice (Figure 6b). Thus, the *p53* and *p21* cell cycle inhibitors have increased expression in Adipo-MROE AT, leading to activation of p66SHC, a downstream target of the p53-p21 pathway.

## 3. Discussion

**Figure 7:**
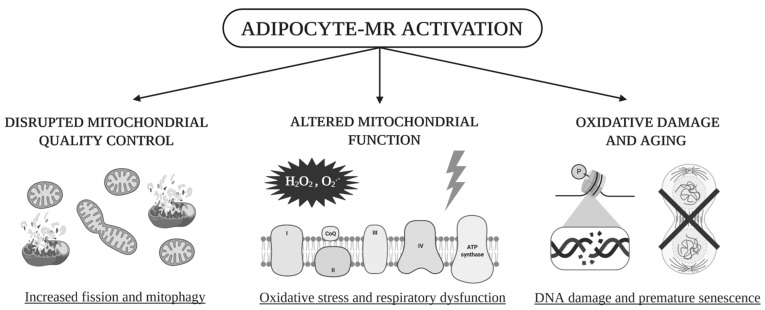
AT MR upregulates mitochondrial fission and mitophagy and inhibits biogenesis and fusion. MR activation in adipocytes (i) disrupts MQC through increased fission and mitophagy and decreased fusion and biogenesis, compromising mitochondrial integrity; (ii) alters mitochondrial respiration and increases mitochondrial OS; as a result, (iii) causes oxidative damage and senescence, leading to AT dysfunction and senescence in obesity. Created with BioRender.com.

In this study, we showed that adipocyte-MR overexpression leads to increased systemic and local OS in AT. This is associated with mitochondrial dysfunction in terms of decreased leak respiration, maximal ETC capacity and reserve capacity and with OS-induced DNA damage. MQC, including biogenesis, dynamics and mitophagy, is impaired as well, leading to reduced mitochondrial mass and a shift towards mitochondrial fission. As a consequence, cellular senescence is increased, promoting the premature ageing of AT. These results are summarized in Figure 7.

Several clinical studies suggest an important role of the renin-angiotensin-aldosterone system in the pathophysiology of obesity and the MetS. The prevalence of hyperaldosteronemia is increased in patients with MetS and vice versa [5,6,41]. This might be due to the ability of lipoproteins and of adipocyte-derived factors, such as leptin, to induce the adrenal synthesis of aldosterone [42,43,44,45]. In patients with primary aldosteronism, treatment through pharmacological MR antagonists (MRA) (eplerenone or spironolactone) or adrenalectomy reduces visceral fat mass [46] and improves systemic and organ insulin sensitivity [47], but other studies have shown only limited effects of MRA in obese diabetic patients [48,49,50]. The reason for these diverging results could be that MRA are often used as a preventive treatment in mice, while human studies are done in patients that are already suffering from the MetS. Adipocyte-MR is overexpressed in both human and mice visceral AT and has been identified as a trigger of OS and insulin resistance [9,51]. Interestingly, our Adipo-MROE model presents a MetS-like phenotype with increased fat mass, insulin resistance, dyslipidemia and hyperglycemia, supporting a causal role of adipocyte-MR in the MetS [9], but the underlying mechanisms remained unclear, hence the present study. 

Increased OS and mitochondrial dysfunction are common AT features in obesity and the MetS [15,52,53,54,55]. In diabetic humans and mice, mitochondrial mass and respiratory chain activity are decreased both in visceral and in subcutaneous AT [56,57,58]. We recently showed that AT mitochondrial dysfunction is MR-dependent in obese db/db mice [17]. However, given the systemic route of administration, it was difficult to delineate the role of adipocyte-MR in this phenotype. Our present data show that adipocyte-MR induces impairment of MQC through reduced biogenesis and increased mitophagy and fission, and alters mitochondrial respiration fitness by reducing maximal ETC capacity and reserve capacity. Reduced maximal capacity of the ETC, and therefore, reduced reserve capacity indicates that when requiring more energy, the mitochondria from MROE-mice cannot match an increased need for ATP phosphorylation with increased electron transfer. The observed reduced leak respiration, i.e., reduced permeability of the mitochondrial membrane to protons, may be a compensatory mechanism to preserve as much ATP phosphorylation as possible, as indicated by the unchanged RCR. These data support a novel molecular mechanism linking adipocyte-MR and AT mitochondrial dysfunction. ETC complex subunit gene expression levels reflect ETC fitness. In particular, complex IV dysfunction is involved in progressive white adipocyte enlargement during aging [59], and the reduction in *Tfam* expression in adipocytes decreases the expression of genes in complexes I and IV, leading to adipocyte dysfunction in visceral white AT [60]. When we assessed gene expression levels of subunits composing these complexes, we observed decreased gene expression. Thus, it seems that ETC dysfunction, by generating OS, is impairing respiration and damaging mitochondria.

These results echo findings made in the context of renal dysfunction. In a renal cell line, aldosterone triggered mitochondrial ROS production and alteration of MQC [25]. This effect was also observed in vivo: aldosterone infusion during 2 weeks increased mitochondrial OS, as well as mitochondrial dysfunction in renal glomerular podocytes, reduced mtDNA copy number and decreased *Tfam* and *Pgc1-α* mRNA levels [25]. In addition, in an animal model of aldosterone-induced nephropathy, inhibition of DRP1 suppressed aldosterone-induced podocyte injury by downregulating mitochondrial fission, mitochondrial dysfunction, and podocyte apoptosis [26]. Mitophagy is another important mechanism involved in MQC. Mitophagy modulation in AT regulates browning and mitochondrial mass [61]. We now report that adipocyte-MR impacts white AT mitophagy.

While ROS are crucial signalling molecules involved in host defense, cell proliferation and metabolic adaptation, a persistent excess of ROS can cause damage to nucleic acids, proteins and lipids, leading to cellular dysfunction and premature ageing. When rats were infused with aldosterone for 5 weeks, MR activation induced senescence in the kidneys, characterised by increased senescence-associated β-galactosidase activity and p53 and p21 expression, as well as decreased SIRT1 expression [62]. These effects were independent from the diuretic effect of aldosterone as they were not observed after hydralazine treatment. p53 activation has multiple consequences for AT function, including: progenitor cell senescence, beigeing inhibition [63], adipogenesis alteration, as well as insulin signalling impairment [28]. p53 can induce apoptosis through permeabilisation of the mitochondrial outer membrane [64]. Aldosterone dose-dependently induced p53 expression and knockdown of p53 inhibited aldosterone-induced DRP1 expression and podocytes injury, suggesting that p53 may mediate aldosterone-induced mitochondrial fission via DRP1 phosphorylation [26]. These results are highly similar to what we report in AT in the present study. In obese db/db mice, we previously observed an MR-dependent upregulation of the p53-p21-p66SHC pathway, as well as an MR-independent increase in p16 mRNA levels, which is consistent with what we observe here in Adipo-MROE mice [17]. SIRT1 is able to inhibit the p53-p21 pathway and its downstream target p66SHC [65]. SIRT3, a mitochondrial sirtuin, regulates fatty acid oxidation, the citric acid cycle and OS response [66]. Thus, reduced sirtuins levels in our model are consistent with the premature senescence we observe. 

In conclusion, adipocyte-MR overexpression is sufficient to induce a MetS-like phenotype, mitochondrial dysfunction and senescence and mimics the increased adipocyte-MR expression we reported in obese patients [9]. However, other cell types are involved in AT dysfunction in the MetS, for example, macrophages. Since macrophages also express MR, further studies should analyse the specific contribution of macrophage MR in AT dysfunction, systemic insulin resistance and diabetes. Moreover, studying the contribution of MR to AT fibrosis, a well-known feature of AT dysfunction could be interesting, as it has already been established in other organs [18,67,68,69]. Taken together, this study, and previous reports, suggest that MR antagonism could be part of the therapeutic arsenal to fight obesity and the MetS [70,71,72]. 

## 4. Materials and Methods

### 4.1. Animal Model

#### 4.1.1. Transgenic Model

The Adipo-MROE mouse model with conditional MR overexpression in adipocytes was generated as previously described [9]. Briefly, the aP2-rtTA mouse strain was crossed with the tetO-hMR mouse strain to generate aP2-rtTA/tetO-hMR (Adipo-MROE) mice, allowing inducible adipocyte-specific MR overexpression upon doxycycline (dox) administration. Transgene expression was induced in 8-week-old mice, matched for age and weight, by administration of 2 g/L of dox in drinking water for 4 weeks (#D3447 Sigma-Aldrich, Saint Quentin Fallavier, France). Littermates (i.e., mono transgenic, tetO-hMR or aP2-rtTA, or wild type) were used as controls (CTRL) and received dox like Adipo-MROE mice. 

#### 4.1.2. Animal Experiments

Animals were kept in a room lighted 12 h per day (7 a.m.–7 p.m.) at a temperature of 22 °C and an average relative humidity of 40%, with *ad libitum* access to a regular chow diet (SAFE number, A04, 2791 kcal/kg, 10% lipids, 67% carbohydrates, and 23% proteins; Augy, France) and tap water. All animal studies were conducted in accordance with the National Institutes of Health Guide and European Community directives for the Care and Use of Laboratory Animals (European Directive, 2010/63/UE) and approved by the local animal ethics committee (agreement #4487-2016010615418571-5). The exclusion criteria were as follows: excessive weight loss (>10%, no animal excluded), ulcerative dermatitis (no animal excluded). Sample size was determined based on previous studies. Because estrogens can influence MR activation [73], male mice only were used in these experiments. The number of mice was 6–10 per group.

#### 4.1.3. Sacrifice and Organ Collection

Sacrifice occurred 12 weeks after the start of the induction. Animals were euthanised in the morning (8 a.m.–12 a.m.). They were anaesthetised with ketamine 100 mg/kg (Kétamine^®^ 1000 Virbac, Carros, France) and xylazine 10 mg/kg (Rompun 2%, Bayer, Vienna, Austria) and the efficacy of anaesthesia was checked by hind leg toe pinching. Mice were then euthanised by cervical dislocation and organs dissected and used for functional studies or stored frozen for further analysis.

### 4.2. Mitochondrial Functional Studies

#### 4.2.1. Mitochondria Isolation

Mitochondria from EVAT were isolated following a previously described procedure [17]. Briefly, EVAT were minced and homogenised in ice-cold isolation buffer (in mmol/L: 250 sucrose, 10 HEPES, pH 7.4, 300 mOsm/kg H_2_O) with a prechilled Potter–Elvehjem homogeniser (400 rpm). Successive centrifugations in a density gradient buffer (in mmol/L: 250 sucrose, 10 HEPES, 3 mg/mL BSA, pH 7.4, 300 mOsm/kg H_2_O) allowed isolation and pelleting of mitochondria. Pellets were kept on ice in assay buffer (in mmol/L: 100 KCl, 20 TES, 4 KH_2_PO_4_, 2 MgCl_2_, 1 EDTA, pH 7.2, 300 mOsm/kg H_2_O) and used for experiments related to oxygen consumption within 4 h. All reagents were purchased from Sigma-Aldrich, Saint-Quentin-Fallavier, France.

#### 4.2.2. Measurement of In Vitro Oxygen Consumption by High Resolution Respirometry

Oxygen consumption in isolated mitochondria was measured with the Oroboros Oxygraph-2k respirometer (O2k, Oroboros Instruments, Innsbruck, Austria). State 4 leak respiration was induced with glutamate and malate (10 mmol/L final concentration). State 3 maximal coupled respiration was induced by adding 300 mmol/L ADP. The involvement of uncoupling proteins (UCPs) was evaluated in state 4 with the specific UCP inhibitor guanosine diphosphate (GDP, 5 mmol/L). UCP-related respiration was calculated as the difference between state 4 leak respiration with and without GDP. Maximal ETC capacity was measured after adding FCCP, a chemical uncoupler (100 nmol/L, titrated to optimal concentration). Reserve capacity was calculated as the difference between maximal ETC capacity and state 3 maximal coupled respiration. Respiratory control ratio was calculated as the ratio between state 3 and state 4 respiration. All measurements were corrected for protein concentration. All reagents were purchased from Sigma-Aldrich, Saint-Quentin-Fallavier, France.

### 4.3. Molecular Studies

#### 4.3.1. Hydrogen Peroxide Concentration Measurement

Hydrogen peroxide concentrations in plasma, EVAT protein lysate and isolated mitochondria were measured using the Amplex™ Red Hydrogen Peroxide/Peroxidase Assay Kit (ref. A22188, Invitrogen™, Villebon-sur-Yvette, France) and following the manufacturer’s guidance. For protein lysates and isolated mitochondria, results were normalised over protein concentration.

#### 4.3.2. Superoxide Concentration Measurement

Superoxide levels were determined by electron paramagnetic resonance (EPR), which allows direct detection of unpaired electrons. In this technique, a spin trapping reagent leads to the formation of a more stable free radical that can be examined by EPR [74]. First, mitochondria isolation was performed as described above. Mitochondrial protein concentrations were measured using the Folin–Lowry method and further allowed normalisation of EPR results. EPR Krebs HEPES buffer was prepared with 25 µM deferoxime (DF) and 5 µM diethyldithiocarbamic acid (DETC). Spin probe Krebs HEPES buffer was prepared with 1 mM 1-Hyroxy-3-carboxy-2,2,5,5-tetramethylpyrrolidine (CPH) to allow detection of superoxide ions. The samples were prepared as follows: the 100 µg of isolated mitochondria were used for two sets of samples with and without 10 µM N-acetylcysteine (NAC, 10 µL) to confirm ROS specific sensitivity. A total of 50 µL of spin probe Krebs HEPES buffer was added to each sample, as well as EPR Krebs HEPES buffer q.s. 1 mL. Samples were incubated for 10 min at 37 °C, snap frozen in a 1 mL syringe in liquid nitrogen, and stored at −80 °C until measurements. Measurements were done with an E-scan (Bruker, Germany) coupled with a temperature controller (Noxygen, Germany) set at 37 °C to mimic in vivo conditions. Comparison of ROS concentrations with and without NAC allowed confirmation of the specific detection of superoxide ions. Values were normalised using protein concentrations and as a percentage of the control. All reagents were purchased from Sigma-Aldrich, Saint-Quentin-Fallavier, France.

#### 4.3.3. 8-OHdG Concentration Measurement

8-OHdG concentrations in plasma were measured using the DNA/RNA Oxidative Damage (High Sensitivity) ELISA Kit (ref. 589320, Cayman Medical, distributed by Bertin Technologies, Montigny-le-Bretonneux, France) and following the manufacturer’s guidance.

#### 4.3.4. Quantitative Real-Time Polymerase Chain Reaction

Total RNA was extracted from EVAT of Adipo-MROE and control mice with TRIZOL reagent (Life Technologies, Carlsbad, USA) according to the manufacturer’s instructions as previously described [17]. The reverse transcription was performed with 2 μg of RNA and the M-MLV Reverse Transcriptase Kit (Life Technologies). Transcript levels of genes were analysed by real-time polymerase chain reaction (fluorescence detection of SYBR green) in duplicate for each sample using an iCycler iQ apparatus (Bio-Rad, Marnes-la-Coquette, France). The primers used were designed using the software Primer3web (version 4.1.0), produced by Eurogentec, Seraing, Belgium, and are listed in Table S1. Importin 8 (Ipo8), being stable in obese animals AT, was used as the reference housekeeping gene for normalisation [75]. The relative copy number of the target genes was calculated with the 2^−ΔΔCt^ method, after assessment that PCR efficiency was 100%. For mtDNA copy number measurement, genomic DNA was extracted using QIAamp DNA Mini Kit (QIAgen, Courtaboeuf, France) and the mtDNA specific gene *ND2* was amplified and normalised over the expression of the nuclear DNA specific gene 18 s. The primers used were designed using the software Primer3 and are listed in Table S2.

#### 4.3.5. Western Blot Analysis

EVAT of Adipo-MROE and control mice were lysed for Western blotting as previously described [17]. Western blot samples were prepared with Laemmli Buffer (BioRad, Marnes-la-Coquette, France) and Criterion TGX Precast Gels (BioRad) were loaded with 30 µg of protein per well. Transfer was done on nitrocellulose membranes using the Trans-Blot^®^ Turbo™ Transfer System (BioRad). Membranes were incubated overnight with primary antibodies (1:1000 in Bovine Serum Albumin 3%, 4 °C), followed by 2 h with horseradish peroxidase-conjugated secondary antibodies (1:4000 in 5% skimmed milk, room temperature). Antibodies used are listed in Table S3. Signals were detected by chemiluminescence (Amersham^TM^ ECL^TM^, GE Healthcare, Buc, France), followed by signal acquisition with the ImageQuant^TM^ LAS 4000 (Fujifilm, Tokyo, Japan). Blots were analysed densitometrically with ImageJ (ImageJ 1.43u). Quantification of phosphorylated proteins was normalised over total protein amount. β-actin was used as a housekeeping protein (1:4000 in Bovine Serum Albumin 3%, 4 °C), or the mitochondrial protein COX4 for TOM20 quantification to prevent variations due to mitochondrial mass. All reagents, otherwise indicated, were purchased from Sigma-Aldrich, Saint-Quentin-Fallavier, France.

### 4.4. Data Analysis

Data are presented as violin plots with quartiles and individual dots. Differences between groups were assessed with the Student’s *t*-test, as appropriate. Values of *p* < 0.05 were considered significant. Statistical analysis and graphs design were performed with GraphPad Prism (version 8.0.1). Researchers were blinded for experimental groups during experimentation and data analysis. The datasets generated and analysed during the current study are available from the corresponding author on reasonable request.

## 5. Conclusions

- Our data highlight the impact of visceral AT adipocyte-MR on mitochondrial function and quality.
- Our work explores mechanisms by which AT mitochondrial quality becomes compromised by MR activation, and how such perturbations alter mitochondrial function.
- Our results support promising therapeutic avenues using MR antagonism as an additive strategy to improve AT mitochondrial function in the context of MetS.

## Data Availability

Researchers were blinded for experimental groups during experimentation and data analysis. The datasets generated and analysed during the current study are available from the corresponding author on reasonable request.

## Supplementary Materials

The following are available online at https://www.mdpi.com/1422-0067/22/6/2881/s1, Supplementary Figure S1: Mineralocorticoid receptor overexpression decreases expression of electron transfer chain sub-units from complexes I and IV; Table S1: Forward and reverse primers sequences used for qRT-PCR on cDNA; Table S2: Forward and reverse primers sequences used for qRT-PCR on genomic DNA; Table S3: Antibodies used for Western Blot membranes hybridisation.
