# Supplementary material for: Adipocyte-Mineralocorticoid Receptor Alters Mitochondrial Quality Control Leading to Mitochondrial Dysfunction and Senescence of Visceral Adipose Tissue
Source: Int J Mol Sci. 2021 Mar 12;22(6):2881. doi: 10.3390/ijms22062881 (PMC8001019; doi:10.3390/ijms22062881)
Supplement: Supplementary file 1 [file ijms-22-02881-s001.pdf]

Adipocyte-Mineralocorticoid Receptor Alters Mitochondrial Quality Control Leading to Mitochondrial Dysfunction and Senescence of Visceral Adipose Tissue - <u>Supplemental materials</u>

Clara LEFRANC[1], Malou FRIEDERICH-PERSSON[2], Fabienne FOUFELLE[1], Aurelie NGUYEN DINH CAT[1†], Frédéric JAISSER[1, 3†].

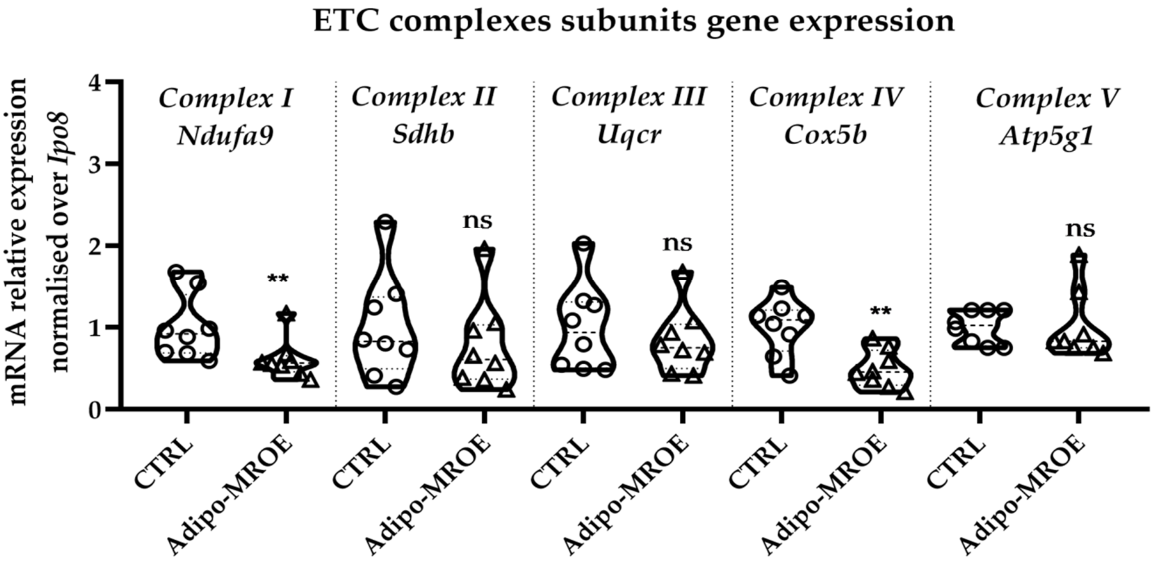

<u>Supplementary Figure 1: Mineralocorticoid receptor overexpression decreases expression of electron transfer chain sub-units from complexes I and IV.</u>

Ipo8: importin 8; Ndufa9: NADH: Ubiquinone Oxidoreductase Subunit A9; Sdhb: succinate dehydrogenase complex iron sulfur subunit B; Uqcr: ubiquinol-cytochrome c reductase complex subunit; Cox5b: Cytochrome c oxidase subunit 5B; Atp5g1: ATP Synthase Membrane Subunit C Locus 1. ** $p<0.01$, ns: non significant Adipo-MROE vs. Ctrl.

Gene	**Forward primer (5′-3′)**	**Reverse primer (5′-3′)**
Atp5g1	AGTTGGTGTGGCTGGATCA	CCCAGAATGGCATAGGAGAA
Cox5b	GGCTGGAGAGGGAGATCATG	TTCACAGATGCAGCCCACTA
Ipo8	GGTCAAACAGGAAGACAGACGTA	CACACCCAAGAACAAGCACA
Lcn2	GGACCAGGGCTGTCGCTACT	GGTGGCCACTTGCACATTGT
Ndufa9	ACTGTGTTTGGGGCTACAGG	GTCACCCATCAGACGAAGGT
p16	GAACTCGAGGAGAGCCATCTG	CTACGTGAACGTTGCCCATC
p21	GTGGTGGAGACCTGATGATACC	CACGGGACCGAAGAGACAAC
p53	CAAGATCCGCGGGCGTAA	GAGGGATGAAGTGATGGGAGC
Pgc1α	GAATCAAGCCACTACAGACACCG	CATCCCTCTTGAGCCTTTCGTG
Ptgds	CTTCCAGCAGGACAAGTTCC	CGGGTCTCACACTGGTTTTT
Sdhb	AGCTACTGGTGGAACGGAGA	GCAGCGGTAGACAGAGAAGG
Tfam	CCGAAGTGTTTTTCCAGCAT	GGCTGCAATTTTCCTAACCA
Uqcr	TGCTGAGCAGGTTTCTAGGC	ATGTAAGGCACCCAGTCCAG

Table S1: Forward and reverse primers sequences used for qRT-PCR on cDNA

Gene	**Forward primer (5′-3′)**	**Reverse primer (5′-3′)**
ND2	CCTATCACCCTTGCCATCAT	GAGGCTGTTGCTTGTGTGAC
18s	CGCCGCTAGAGGTGAAATTC	TCTTGGCAAATGCTTTCGC

Table S2: Forward and reverse primers sequences for qRT-PCR on genomic DNA

Antibody	Protein	Reference / antibody type	Dilution
Primary	MFN-2	CST D2D10 #9482 Monoclonal rabbit	1/1000
	DRP-1	CST D6C7 #8570 Monoclonal rabbit	
	Phospho-DRP1	CST Ser616 #3455 Polyclonal rabbit	
	SIRT1	CST #2028 Polyclonal rabbit	
	SIRT3	CST #5490 Monoclonal rabbit	
	H2A.X	CST # 2595 Polyclonal rabbit	
	Phospho-H2A.X	CST Ser139 #2577 Polyclonal rabbit	
	COX4	CST 3E11 #4850 Monoclonal rabbit	
	P66-SHC	Merck #06203 Polyclonal rabbit	
	Phospho-P66-SHC	Merck Ser36 #566807 Monoclonal Mouse	
	TOM20	sc-17764 Monoclonal mouse	
	β-actine	Sigma A2228 Monoclonal mouse	1/7500
Secondary	Anti-Rabbit	Dako #P0448 Polyclonal Goat	1/4000
	Anti- Mouse	Dako #P0447 Polyclonal Goat	

Table S3: Antibodies used for Western Blot membranes hybridisation
